# Low incidence of maxillary hypoplasia in isolated cleft palate

**DOI:** 10.1186/s40902-020-00252-9

**Published:** 2020-03-20

**Authors:** Vitali Azouz, Marilyn Ng, Niyant Patel, Ananth S. Murthy

**Affiliations:** 1 Department of Surgery, Summa Health System, 55Arch Street, Suite 2F, Akron, OH 44304-1423 USA; 2 Plastic, Reconstructive and Hand Surgery, Northwell Health-Staten Island University Hospital, Staten Island, NY USA; 3 Plastic & Reconstructive Surgery, Akron Children’s Hospital, Akron, OH USA

**Keywords:** Cleft lip and palate, Maxillary growth, Isolated cleft palate, Complications, Velopharyngeal insufficiency, Palatal fistula

## Abstract

**Background:**

The cause of maxillary growth restriction in patients with cleft lip and palate remains controversial. While studies have investigated the effects surgical technique and timing have on maxillary growth, few focus on patients with isolated cleft palate (ICP). The purpose of this study was to determine the impact palate repair and its associated complications may have on maxillary growth.

**Methods:**

A retrospective chart review of ICP patients who underwent palatoplasty from 1962 to 1999 at Akron Children’s Hospital was performed. Patient demographics, Veau type, age at primary repair, closure technique, presence of fistula or velopharyngeal insufficiency (VPI), number of palatal operations, maxillary hypoplasia (MH) frequency, and follow-up were recorded. Exclusion criteria included patients with cleft lip, submucous cleft, or syndromes.

**Results:**

Twenty-nine non-syndromic ICP patients were identified; 62% (*n* = 18) had Veau type 1 and 38% (*n* = 11) had Veau type 2. All patients underwent 2-flap or Furlow palatoplasty with mobilization of mucoperiosteal flaps. Vomerine flaps were used in all Veau 2 cleft palate closures. Palatoplasty was performed at a mean age of 19.9 ± 8.2 months. Average follow-up was 209 ± 66.5 months. The rate of VPI was 59% (*n* = 17) and the rate of oronasal fistula was 14% (*n* = 4).

**Conclusions:**

There was a low incidence of MH despite complications after initial palate closure. Our results seem to suggest that age at palate closure, type of cleft palate, and type of surgical technique may not be associated with MH. Additionally, subsequent procedures and complications after primary palatoplasty such as VPI and palatal fistula may not restrict maxillary growth.

## Introduction

The cause of maxillary growth restriction in patients with cleft lip and palate (CLP) remains controversial. Some authors suggest that the intrinsic primary anomaly leads to maxillary hypoplasia (MH) [1–3]. Other authors believe that palatoplasty contributes to this phenomenon as numerous reports have noted normal maxillary growth in nonoperative CLP patients [4–9]. This has led to numerous studies that investigated the effects of surgical technique and timing have on maxillary growth. Interestingly, patients who underwent lip repair for cleft lip alone had similar maxillary retrusion rates to CLP patients that underwent lip and palate repair [8, 9] suggesting that lip repair and not palate repair may case growth restriction [8, 10, 11].

Currently, few studies focus on isolated cleft palate (ICP) patients [2, 12–15]. The purpose of this study was to determine the impact palate repair and its associated complications may have on maxillary growth.

## Methods

After obtaining institutional review board approval, a retrospective chart review of non-syndromic, ICP patients who underwent palatoplasty from 1962 to 1999 at Akron Children’s Hospital was performed. Patient demographics, Veau type, age at primary cleft palate repair, repair technique, presence of fistula or velopharyngeal insufficiency (VPI), age at VPI correction, total number of palatal operations, MH frequency, and length of follow-up were recorded for each patient. Patients were determined to have velopharyngeal insufficiency based on perceptual speech evaluation performed by a certified speech and language pathologist. MH was defined as any patient that required maxillary advancement and/or whose occlusion could not be corrected orthodontically. A minimum follow-up of 12 years was used to determine maxillary position. Exclusion criteria included patients with cleft lip, syndromic patients, patients with a submucous cleft, patients who had corrective surgery after 4 years of age, and patients whose age at follow-up was less than 12 years.

Subgroup analysis was performed to determine the relation of age groups to MH. Age group 1 included patients less than 12 months. Age group 2 included patients from 12.1 to 18 months. Age group 3 included patients from 18.1 to 24 months. Age group 4 included patients from 24 to 48 months.

Categorical variables are presented as actual incidence. Continuous variables are presented as means with standard deviations. Categorical variables were examined using the chi-square or Fisher’s exact test as appropriate. Continuous variables were examined using a student’s *t* test. Statistical significance was set at *p* < 0.05. Incomplete charts were excluded from our analysis. Microsoft Excel (Microsoft Corp., Redmond, WA, USA) was used for all analyses.

## Results

**Fig. 1:**
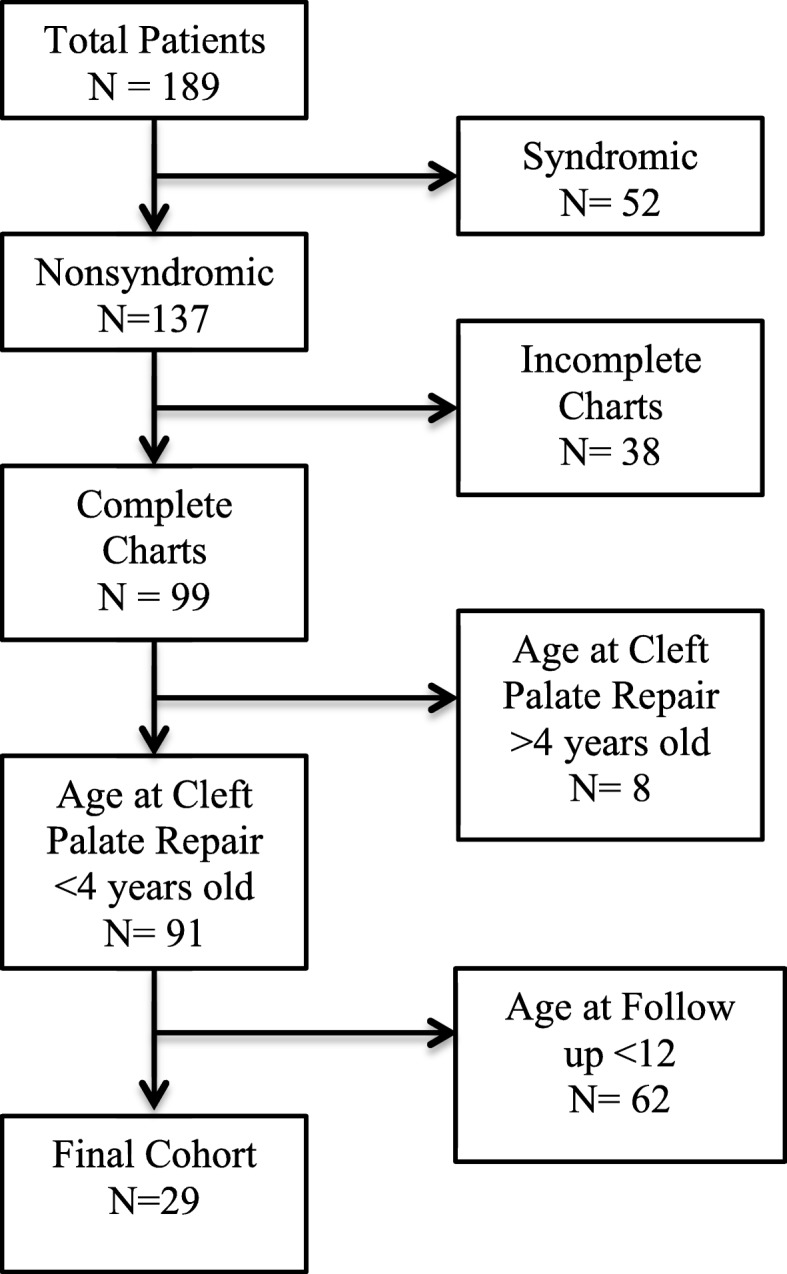
Flow diagram of chart review

Our retrospective review identified 189 patients. After applying our exclusion criteria, 29 non-syndromic ICP patients that underwent surgical repair by 8 surgeons were identified (Fig. 1); 14 patients (48%) were males and 15 patients (52%) were female. Using the Veau classification, 62% (*n* = 18) had Veau type 1 cleft palate and 38% (*n* = 11) had Veau type 2. Mucoperiosteal (Veau) flaps were raised in all palate closures, and vomerine flaps were used in all Veau type 2 cleft palate closure. Palatoplasty was performed at a mean age of 19.9 ± 8.2 months (Table 1). Patients were followed for an average of 209 ± 66.5 months and all patients were older than 12 at follow-up.

**Table 1:** Age and time of surgery (months)

	Mean	Standard deviation	*N*
Age at CP repair	19.8	8.2	29
Age at VPI surgery	147.1	91.2	13
Time from CP to VPI surgery	88.8	61.8	9

*VPI* velopharyngeal insufficiency, *CP* cleft palate

The rate of VPI was 59% (*n* = 17) and the rate of oronasal fistula was 14% (*n* = 4). Fourteen patients required secondary speech surgery and underwent surgery at a mean age of 147.1 ± 91.2 months (Table 1). Of these patients, 79% (*n* = 11) underwent a pharyngeal flap and 7% (*n* = 3) underwent pharyngoplasty. Half of the patients that developed an oronasal fistula had corrective surgery. Subgroup analysis failed to reach statistical significance. Patients in group 2 had the highest incidence of VPI and complications. The only case of MH occurred in group 3 (Table 2). This one patient (2%), who underwent palate closure at 14 months, developed maxillary hypoplasia requiring LeFort I advancement at 16 years. The patient subsequently developed VPI requiring corrective surgery at 17.3 years.

**Table 2:** Subgroup analysis by age

Age group (months)	Number of patients	MH	Fistula	VPI
**Group 1 < 12**	4	0	2	3
**Group 2 > 12–18**	13	0	1	9
**Group 3 > 18–24**	6	1	1	3
**Group 4 > 24**	6	0	0	2

*VPI* velopharyngeal insufficiency, *CP* cleft palate, *MH* maxillary hypoplasia

## Discussion

Since noted by Graber [16], the cause of maxillary growth in CLP patients remains controversial to this day. In our study, we exclusively reviewed ICP patients with Veau types 1 and 2 that underwent surgical repair in our study to determine factors associated with MH. Though the low incidence of MH in our study is similar to that found in the literature [14, 17, 18], our small sample size prevented statistical analysis. However, our results seem to suggest that the age of initial palatal closure and palatoplasty technique are not associated with maxillary growth restriction. Multiple surgeons were involved in our study, and in spite of varied techniques, and differing results in terms of fistula and VPI, the variability did not seem to contribute to maxillary growth in ICP.

Numerous studies have shown the adverse effects of scarring after palatoplasty [5–7, 9] and cheiloplasty have on maxillary growth [8, 10, 11]. In our study, we only evaluated ICP patients to isolate the potential effect that palate repair may have on maxillary growth. Only one patient in our cohort developed MH despite a high rate of postoperative complications including VPI and oronasal fistula. This seems to suggest that postoperative complications and the cumulative number of subsequent surgical procedures that address these complications do not contribute to maxillary growth restriction. This may imply that scaring from the initial palatoplasty and subsequent procedures does not affect maxillary growth restriction. Although many authors have focused on the relationship between surgical technique of the initial palatoplasty and maxillary growth [19–23], we believe technical nuances such as raising mucoperiosteal flaps or vomerine flaps may not alter the maxillary growth trajectory. Patient age at the time of repair also did not seem to have an effect either. Our average age at initial palatoplasty exceeded that of the patient requiring MH (19 vs. 14 months). These observations are similar to that of Odom et al. who also noted that timing and techniques for palatal repair do not have a deleterious effect on antero-posterior maxillary growth [14].

The high rate of secondary procedures we observed in our cohort despite a low incidence of MH is also noteworthy. Interestingly, a higher rate of secondary procedures has been reported after cleft palate closure in patients with ICP. As Chorney et al. noted in their case series of 312 patients, patients with Veau type 2 have a statistically higher rate of pharyngeal flap requirement and oronasal fistula repair [21]. Similarly, in their study of 869 non-syndromic cleft palate patients, Jackson et al. found that patients with ICP had the worst speech outcomes and higher rates of secondary surgery for velopharyngeal dysfunction [19]. In our cohort, four patients developed oronasal fistulas and one of these patients required two corrective procedures. This patient as well as others who had multiple corrective surgeries for complications had no evidence of maxillary growth restriction at follow-up. Seventeen patients developed VPI and 4 of these patients required at least 2 revisionary surgeries. Subgroup analysis seemed to suggest that patients whose cleft was repaired prior to 12 months were more likely to develop palatal fistulae, and overall complications where patients whose clefts were repaired between 12 and 18 months had an increased risk to develop VPI. The majority of our patients had their initial corrections performed by non-fellowship trained surgeons prior to 1980. This may explain our high complication rate as evolving surgical techniques significantly improved outcomes after palate repair thereafter. However, in spite of our high reported VPI and fistula rate, we observed a low incidence of MH. Our results seem to suggest that age at palate closure, type of cleft palate (Veau 1 vs. 2) and type of surgical technique may not be associated with MH. Additionally, complications after primary palatoplasty such as VPI and palatal fistula and subsequent procedures may also not restrict maxillary growth.

Due to our low incidence of MH, our ability to deduce statically significant conclusions was limited. Additionally, our study was limited by its retrospective nature and relied on available charting. In our study, MH was defined by clinical judgment to perform LeFort I osteotomy and lacked quantitative lateral cephalograms that may have allowed more objective assessment and comparison to other studies. Larger prospective studies are needed to further evaluate the association of surgical technique and the rate of MH in the ICP patient population.

## Conclusions

There was a low incidence of MH despite complications after initial palate closure. Our results seem to suggest that age at palate closure, type of cleft palate, and type of surgical technique may not be associated with MH. Additionally, subsequent procedures and complications after primary palatoplasty such as VPI and palatal fistula may not restrict maxillary growth.

## Data Availability

Data may be made available upon request.
